# Supplementary material for: A cautionary tale about properly vetting datasets used in supervised learning predicting metabolic pathway involvement
Source: PLoS One. 2024 May 2;19(5):e0299583. doi: 10.1371/journal.pone.0299583 (PMC11065254; doi:10.1371/journal.pone.0299583)
Supplement: Attachment — Submitted filename: response_to_r1_reviewer_comments_v3.docx [file pone.0299583.s001.docx]

**Journal requirements:**

*When submitting your revision, we need you to address these additional requirements.*

**Response:**

We have addressed all of these requirements.

**Issue 1:**

*1. Please ensure that your manuscript meets PLOS ONE's style requirements, including those for file naming. The PLOS ONE style templates can be found at*

[*https://journals.plos.org/plosone/s/file?id=wjVg/PLOSOne_formatting_sample_main_body.pdf*](https://journals.plos.org/plosone/s/file?id=wjVg/PLOSOne_formatting_sample_main_body.pdf) *and*

[*https://journals.plos.org/plosone/s/file?id=ba62/PLOSOne_formatting_sample_title_authors_affiliations.pdf*](https://journals.plos.org/plosone/s/file?id=ba62/PLOSOne_formatting_sample_title_authors_affiliations.pdf)

**Response:**

We believe the manuscript conforms to *PLOS One*’s style requirements.

**Issue 2:**

*2. Please note that PLOS ONE has specific guidelines on code sharing for submissions in which author-generated code underpins the findings in the manuscript. In these cases, all author-generated code must be made available without restrictions upon publication of the work. Please review our guidelines at* [*https://journals.plos.org/plosone/s/materials-and-software-sharing#loc-sharing-code*](https://journals.plos.org/plosone/s/materials-and-software-sharing#loc-sharing-code) *and ensure that your code is shared in a way that follows best practice and facilitates reproducibility and reuse.*

**Response:**

All data, code, and results are available in the following Figshare item: <https://doi.org/10.6084/m9.figshare.22661185>

**Issue 3:**

*3. Thank you for stating the following financial disclosure:*

*“This work has been supported by the National Science Foundation [NSF 2020026 to HNBM]. “*

*Please state what role the funders took in the study.  If the funders had no role, please state:* *"The funders had no role in study design, data collection and analysis, decision to publish, or preparation of the manuscript."*

*If this statement is not correct you must amend it as needed.*

*Please include this amended Role of Funder statement in your cover letter; we will change the online submission form on your behalf.*

**Response:**

The funders had no role, so please use the following statement:

"The funders had no role in study design, data collection and analysis, decision to publish, or preparation of the manuscript."

**Issue 4:**

*4. Thank you for stating the following in the Acknowledgments Section of your manuscript:*

*“This work has been supported by the National Science Foundation [NSF 2020026 to HNBM].  We thank Dr. Robert Flight for feedback on the machine learning evaluation methodology utilized in this work. We thank the University of Kentucky Center for Computational Sciences and Information Technology Services Research Computing for their support and use of the Lipscomb Compute Cluster and associated research computing resources.”*

*We note that you have provided funding information that is currently declared in your Funding Statement. However, funding information should not appear in the Acknowledgments section or other areas of your manuscript. We will only publish funding information present in the Funding Statement section of the online submission form.*

*Please remove any funding-related text from the manuscript and let us know how you would like to update your Funding Statement. Currently, your Funding Statement reads as follows:*

*“This work has been supported by the National Science Foundation [NSF 2020026 to HNBM]. “*

*Please include your amended statements within your cover letter; we will change the online submission form on your behalf.*

**Response:**

We have removed the funding information from the Acknowledgement section.

**Issue 5:**

*5. Please review your reference list to ensure that it is complete and correct. If you have cited papers that have been retracted, please include the rationale for doing so in the manuscript text, or remove these references and replace them with relevant current references. Any changes to the reference list should be mentioned in the rebuttal letter that accompanies your revised manuscript. If you need to cite a retracted article, indicate the article’s retracted status in the References list and also include a citation and full reference for the retraction notice.*

**Response:**

We have checked the references.

**Reviewer #1:**

*This article A cautionary tale about properly vetting datasets used in supervised learning predicting metabolic pathway involvement is structured properly authors did the statistical analysis. But some changes they have to do these are.*

**Response:**

We thank the reviewer for the positive comments. We have addressed each issue raised.

**Issue 1:**

*1.proof reqd the entire manuscript*

**Response:**

We have proofread the entire manuscript. But if we have still missed anything, please let us know.

**Issue 2:**

*2) Authors should draw graphical abstract*

**Response:**

It is quite hard to create a graphical abstract for a paper with no figures. But we have made a try at creating a graphical abstract that describes data leakage, which is the central problem we detected.

**Issue 3:**

*3) Compare your study am with existing systems*

**Response:**

With all due respect, there is nothing to compare to. We detected a catastrophic data leak in the results generated for 3 papers. The authors of the first paper have acknowledged the problem with a comment on our preprint and said they are publishing a correction to their flawed paper:

Erik D. Huckvale and Hunter N.B. Moseley. "A cautionary tale about properly vetting datasets used in supervised learning predicting metabolic pathway involvement" ***bioRxiv*** 2023.10.03.560711 (2023). <https://doi.org/10.1101/2023.10.03.560711>

We have also published a sister paper demonstrating a properly created dataset and classification results using 3 different machine learning methods:

Erik D. Huckvale, Christian D. Powell, Huan Jin, and Hunter N.B. Moseley. "Benchmark dataset for training machine learning models to predict the pathway involvement of metabolites" ***Metabolites*** 13, 1120 (2023). <https://doi.org/10.3390/metabo13111120>

**Reviewer #2:**

*The manuscript entitled “A cautionary tale about properly vetting datasets used in supervised learning predicting metabolic pathway involvement” has many mistakes, authors need to rectify many portions.*

**Response:**

Respectfully, we disagree. Most of the issues and questions raised by the reviewer are asking for more detail and clarification, but do not identify mistakes in our manuscript. We have addressed these issues by adding more detail and clarification, when warranted. The authors of Baranwal et al have already acknowledged the data leakage problem in the dataset they generated in a comment posted on our preprint:

Erik D. Huckvale and Hunter N.B. Moseley. "A cautionary tale about properly vetting datasets used in supervised learning predicting metabolic pathway involvement" ***bioRxiv*** 2023.10.03.560711 (2023). <https://doi.org/10.1101/2023.10.03.560711>

**Issue 1:**

*Should there be more precision in explaining the machine learning applications, such as the specific methodologies used by Hu et al, Baranwal et al, Yang et al, and Du et al?*

**Response:**

Our manuscript is focused on the catastrophic data leakage in the dataset and its effects on model evaluation. The specific methodologies used in the machine learning methods is secondary. However, our manuscript describes what these methods are:

“The first of these machine learning applications was Hu et al [4] which used a random forest (RF) model [5] to predict metabolic pathway involvement, training a model to assign compounds to one or more of eleven KEGG pathways (see the ‘Included As A Label In The KEGG-SMILES Dataset’ column in Table 1). The 12th label was not included in these publications, likely because it resulted in poor machine learning performance as compared to the other labels [6].

Later publications introduced deep neural networks in an attempt to improve model performance for this machine learning task, including Baranwal et al [7] who reports higher model performance scores using a graph convolutional network (GCN) [8] combined with an RF as well as a GCN by itself. While the model used by Hu et al [4] and the dataset used to train it are not provided in their publication or supplemental material (at least we could not find it), Baranwal et al provides the code they used as well as the dataset they created, as seen in Table 2. This dataset is a text file, with each line containing the associated KEGG pathway labels used for supervised learning preceded by a simplified-molecular-input-line-entry-system (SMILES) [9] representation of the metabolic compound, and is available in their GitHub repository [10]. We will refer to this dataset as the KEGG-SMILES dataset (Table 1). Yang et al [11] claims to further improve on the performance of the model proposed by Baranwal et al [7] using an attention-based [12] graph network.”

**Issue 2:**

*Could there be confirmation or additional details on the machine learning model used by Hu et al [4], especially the dataset they used and its characteristics?*

**Response:**

We are limited to what authors provided in Hu et al [4], which does NOT include their dataset, model, code, or full results. This is a problem in many older machine learning papers that did not follow best practices for supporting computational scientific reproducibility. Please refer to the following paper in *Science* on best practices in publishing computational results:

Stodden V, McNutt M, Bailey DH, Deelman E, Gil Y, Hanson B, Heroux MA, Ioannidis JP, Taufer M. Enhancing reproducibility for computational methods. ***Science***. 2016 Dec 9;354(6317):1240-1.

**Issue 3:**

*Should there be more information on why the 12^th^ label was excluded in the machine learning predictions, and what criteria were used for inclusion or exclusion?*

**Response:**

We can only speculate on why the authors of the prior papers did not include the 12^th^ pathway label.

However, our sister paper highlights a likely reason since the 12^th^ pathway label provided the poorest classification results:

Erik D. Huckvale, Christian D. Powell, Huan Jin, and Hunter N.B. Moseley. "Benchmark dataset for training machine learning models to predict the pathway involvement of metabolites" ***Metabolites*** 13, 1120 (2023). <https://doi.org/10.3390/metabo13111120>

The manuscript mentions this possibility:

“The 12th label was not included in these publications, likely because it resulted in poor machine learning performance as compared to the other labels [6].”

**Issue 4:**

*Should there be confirmation or additional details on the availability of datasets, particularly the KEGG-SMILES dataset, and how researchers can access and utilize it?*

**Response:**

Baranwal et al and Du et al provided their datasets. This is clearly indicated in Table 2 of our manuscript. Also, we have included their datasets in our Figshare item:

<https://doi.org/10.6084/m9.figshare.22661185>

Hu et al and Yang et al did NOT provide their datasets, code, models, or full results, and thus, did not follow best practices in support computational scientific reproducibility.

**Issue 5:**

*Could there be more explanation on the format of the KEGG-SMILES dataset, such as the structure of each line and how the SMILES data is represented?*

**Response:**

The KEGG-SMILES dataset is a simple text file that includes a SMILES string followed by the list of pathway labels as simple numbers. We have added a figure (Figure 1) showing the first few entries in the dataset, along with a basic description of the format.

**Issue 6:**

*Should there be more clarification on how the dataset size varies across different publications, and what factors led to the variations?*

**Response:**

The manuscript clearly present how the dataset varies across different publications in Table 2. The factors for the variations are mention in the manuscript:

“Yang et al [11] claims to further improve on the performance of the model proposed by Baranwal et al [7] using an attention-based [12] graph network. While the authors don’t appear to provide code or data, they evidently used the same dataset to train their model considering the number of instances is the same and they also describe it as containing SMILES data in their manuscript. Finally, Du et al [13] presents the most recent machine learning models generated from this KEGG-SMILES dataset, with the same text file available in their GitHub repository [14]. Their dataset size is slightly smaller than previous publications, because feature vectors could not be generated from all chemical compound structures in the KEGG-SMILES dataset, causing these unconvertable entries to be dropped.”

**Issue 7:**

*Could there be more precision in reporting metrics, especially regarding the accuracy, precision, recall, and F1 score calculations?*

**Response:**

We already include 4 digits of precision, which is likely higher precision than these metrics warrant. We also include standard deviations, which statistically characterizes the variation in these metrics. This is all presented in Tables 3 and 10.

**Issue 8:**

*Should there be an explanation or investigation into the reasons for the large numbers of exact duplicate entries in the KEGG-SMILES dataset, and how it might affect the validity of the results?*

**Response:**

We have already identified a data leakage problem caused by the large number of exact duplicates, which the authors of Baranwal et al have verified exists. At the time we submitted this manuscript, we did not have access to the code that generated the dataset. Given our familiarity with using the KEGG API (please see our kegg_pull publication), we know that certain approaches for using this REST API will likely produce list of entries with duplicates. Also, the authors of Baranwal et al have made updates to their code repository and included a script that generates a dataset via the KEGG API. However upon inspection, this script does not create the original dataset in a tab-delimited kegg_classes.txt file. It creates a CSV file called keggdb2.csv.

**Issue 9:**

*Could there be more information on how the validity of results presented by Baranwal et al [7], Yang et al [11], and Du et al [13] is being questioned, and what specific concerns ?*

**Response:**

It is a data leakage problem arising from a large number of duplicate entries in the dataset. The authors of Baranwal et al have confirmed the problem exists. A large part of the Discussion section is devoted to describing the problem:

“Machine learning researchers such as Zhao et al [20] and Allamanis [21] have touched on the issues in validity that arise when machine learning datasets contain exact duplicates. Their results complement ours (Table 10), showing that machine learning datasets containing duplicates inflate model performance compared to the de-duplicated counterparts. The bias introduced by entry duplicates represents a non-random sampling and can result in overestimating the performance when evaluating machine learning models, considering duplicates occur both in the training and testing sets across CV folds. More generally, the presence of exact duplicates in a dataset is a type of data leakage, where data used in training is leaked into the testing [22] [23]. Therefore, the results from Baranwal et al [10] [7], Yang et al [11], and Du et al [14] [13] have inflated model performances. Based on our results in Table 10. These model performances are most likely inflated by roughly 10% in precision, recall, and F1 score. In the case of the MLGL-MP in particular, we can see in the training/evaluation script of the model that the test set is evaluated in each training epoch: https://github.com/dubingxue/MLGL-MP/blob/main/MLGL-MP-%28fold-1%29/Training.py. The scores reported are those from the epoch that evaluated best, therefore using the test set for model selection. While best practice for machine learning is to fully train the model and evaluate the test set only once per CV iteration, we retained the evaluation methods of Du et al in order to make a maximally accurate comparison.

The standard deviation also increases significantly in the de-duplicated version of the KEGG-SMILES dataset (Table 10), which is expected since the presence of duplicates was artificially increasing performance. However, the standard deviation was calculated from the same 10 CV folds used in each run, which does not provide a reliable estimate of the performance variance since the training and test sets do not change. In fact, it grossly underestimates the real performance variance, since only the stochastic model training process is changing over the 10 iterations per CV fold. But to make an apples-to-apples comparison, we were forced to use the same protocol.”

**Issue 10:**

*Should there be more details on how the scripts were modified to process and train/evaluate the model across all ten cross-validation folds, and how the original repository handled these folds?*

**Response:**

These changes in the processing scripts were minor and only for the purposes of taking either the original KEGG-SMILES dataset or a deduplicated version and to calculate performance metrics across the 10 cross-validation folds. We indicated this as follows in the manuscript:

“Fortunately, the original repo did contain the ten CV folds as CSV files (i.e. a train.csv and test.csv for each fold), so we modified the scripts further in order to process and train/evaluate the model across all ten folds rather than only one.”

“But this dataset deduplication analysis necessitated further minor modification of the original scripts provided by Du et al [14] in order to optionally take in either the KEGG-SMILES dataset containing duplicates or the de-duplicated version. That enabled us to produce model performance results on both the original dataset and de-duplicated dataset.”

Moreover, all of the modified scripts are in the Figshare item:

<https://doi.org/10.6084/m9.figshare.22661185>

**Issue 11:**

*Could there be an explanation for not being able to exactly reproduce the published results due to the lack of seeding in model training? How did running each of the ten folds multiple times contribute to approximating the original results?*

**Response:**

These methods are stochastic and not deterministic. They will NOT produce exactly the same results without setting a random number seed. This is a well-known issue with stochastic methods. But we have made the following revisions to make this point clearer:

“Since these methods are stochastic and the model training was not seeded, we were not able to exactly reproduce the published results.”

**Issue 12:**

*Should there be more details on the training/model evaluation script, especially regarding the parameters used, the role of stochastic gradient descent, and the selection process for reporting scores?*

**Response:**

We now have this paragraph in the ‘Materials and methods’ section that provides more details regarding the optimization method and model training, including the parameter values:

“In the training/model evaluation script of Du et al [[14]](https://sciwheel.com/work/citation?ids=14676257&pre=&suf=&sa=0&dbf=0), the model is trained via stochastic gradient decent over 200 epochs (batch size of 256), specifically using the Adam optimizer [[15]](https://sciwheel.com/work/citation?ids=12703415&pre=&suf=&sa=0&dbf=0). The graph neural network was trained with a dropout layer of probability 0.2. The ReLu activation function was used for all but the final activation, in which a LeakyReLu was used with a negative slope of 0.2. A learning rate of 0.0005 was used for the Adam optimizer along with betas of 0.9 and 0.999, epsilon of 10^-8, and a weight decay of 0. “

The complements the following description of Due et al’s methods:

“we were able to closely approximate the original results by running each of the ten folds ten times (to account for differences in random seed and the stochastic nature of model initialization and training) for a total of 100 runs.”

“or every CV fold, the test set is evaluated in each epoch and the scores reported are those from the epoch where the model performed best on the test set. In other words, the test set is evaluated multiple times and the highest scores are chosen from the multiple evaluations.”

**Issue 13:**

*Should there be more information on the modifications made to Du et al’s original scripts to accommodate either the KEGG-SMILES dataset containing duplicates or the de-duplicated version?*

**Response:**

These changes in the processing scripts were minor and only for the purposes of taking either the original KEGG-SMILES dataset or a deduplicated version and to calculate performance metrics across the 10 cross-validation folds. We indicated this as follows in the manuscript:

“Fortunately, the original repo did contain the ten CV folds as CSV files (i.e. a train.csv and test.csv for each fold), so we modified the scripts further in order to process and train/evaluate the model across all ten folds rather than only one.”

“But this dataset deduplication analysis necessitated further minor modification of the original scripts provided by Du et al [14] in order to optionally take in either the KEGG-SMILES dataset containing duplicates or the de-duplicated version. That enabled us to produce model performance results on both the original dataset and de-duplicated dataset.”

Moreover, all of the modified scripts are in the Figshare item:

<https://doi.org/10.6084/m9.figshare.22661185>

**Issue 14:**

*Could there be more details on the statistics collected and the results derived from the scripts, especially the summary statistics and the nature of the statistical analyses performed?*

**Response:**

The accuracy, precision, recall, and F1-score metrics are standard and well-known in the machine learning field. The statistics we provide are mean and standard deviation of these metrics. This is fully described in Tables 3 and 10 footnotes and in the main text.

**Issue 15:**

*Should there be clarification on why the fractions in Table 5, representing the proportion of compounds in the dataset with a given pathway label, do not add up to one? How does the possibility of entries being associated with more than one pathway label contribute to this?*

**Response:**

Some of the entries have multiple pathway labels. We have expanded this explanation to the manuscript:

“Note the fractions do not add up to one, since some entries are associated with more than one pathway label. Thus, these entries are counted more than once with respect to pathway inclusion.”

**Issue 16:**

*Could there be an explanation of the rationale behind de-duplicating the dataset? How does removing recurring duplicates impact the subsequent analyses?*

**Response:**

Deduplicating the dataset removes the catastrophic data leakage across training and testing phases. This then allows the resulting model performance evaluation to produce valid results. We have added this explanation in the Materials and Methods section:

“The purpose of the deduplication is to allow a valid evaluation of model performance.“

**Issue 17:**

*Should there be more interpretation of the two notable observations in Table 5, especially regarding the higher percentage of duplicates in every pathway category and the lower proportion of the dataset in each pathway category in the de-duplicated dataset?*

**Response:**

This is explained by Tables 7 and 8 showing that duplicate entries are enriched with multiple pathway labels. We have added this explanation with the Table 8 description.

“We see in Table 8 that the non-duplicate subset had a significantly smaller average number of labels and percentage of entries with multiple labels than the original dataset and the subset containing the duplicates had significantly higher values of these same metrics. The ratio of the average number of labels between duplicates and non-duplicates is over 2.5 and the ratio of the percentage of entries with multiple labels between duplicates and non-duplicates is over 20. These differences also explain why the pathway fractions in Table 5 were all lower in the deduplicated dataset vs the original dataset.”

**Issue 18:**

*Could there be more details on the suspected relation between the number of times a unique entry occurs in the original dataset and the number of pathway labels it has? How does Table 6 illustrate this relationship?*

**Response:**

We make these points with Tables 7 and 8, since it is harder to see the pattern in Table 6.

“The resulting contingency table, i.e. Table 7, shows a non-random dependent relationship between the number of occurrences versus the number of labels, with the top left to bottom right diagonal weighted in the 2x2 table.”

“Unlike the metrics from Table 6 and Table 7, we include all the entries as they are in the original dataset, i.e. instead of unique entries being counted once, each recurrence of a duplicate is included as well. We see in Table 8 that the non-duplicate subset had a significantly smaller average number of labels and percentage of entries with multiple labels than the original dataset and the subset containing the duplicates had significantly higher values of these same metrics.”

**Issue 19:**

*Should there be clarification on how Table 6 is compressed to create Table 7? What does the resulting contingency table reveal about the relationship between the number of occurrences and the number of labels?*

**Response:**

We believe there is a clear description of this in the manuscript:

“Table 6 can be compressed along the rows into two categories: the top row representing the unique entries that only occur once and the remaining rows representing the unique entries that occurred more than once. The columns can be compressed into two categories with the first column representing entries with just one pathway label and the remaining columns being those with multiple labels. The resulting contingency table, i.e. Table 7, shows a non-random dependent relationship between the number of occurrences versus the number of labels, with the top left to bottom right diagonal weighted in the 2x2 table.”

**Issue 20:**

*Could there be more information on the significance of the metrics in Table 8, especially the average number of labels and the percentage of entries with multiple labels? How do these metrics differ between the non-duplicate subset, the original dataset, and the subset containing duplicates?*

**Response:**

We have expanded the description of Table 8 as follows:

“We see in Table 8 that the non-duplicate subset had a significantly smaller average number of labels and percentage of entries with multiple labels than the original dataset and the subset containing the duplicates had significantly higher values of these same metrics. The ratio of the average number of labels between duplicates and non-duplicates is over 2.5 and the ratio of the percentage of entries with multiple labels between duplicates and non-duplicates is over 20. These differences also explain why the pathway fractions in Table 5 were all lower in the deduplicated dataset vs the original dataset.”

**Issue 21:**

*Should there be more context on the statistical tests performed in Table 9, such as the Chi Square test, Fisher's Exact test, and Mann-Whitney U test? How do these tests contribute to the overall interpretation of the results?*

**Response:**

We performed 3 different statistical tests so that there would be no argument about statistical significance. We even added an explanation about the effect sizes. We believe the following is more than adequate:

“The Chi Square test of the contingency table (Table 6) resulted in a p-value of 0. Since the compressed contingency table (Table 7) had two rows and two columns, we used a Fisher’s Exact test resulting in a p-value of 8.38x10^-254. Additionally, we performed a Mann-Whitney U test comparing the number of labels of non-duplicate entries to the number of labels of duplicate entries (Table 8) resulting in a p-value of 0. While the effect sizes of the chi-squared (Cramer V= 0.2995) and Mann Whitney U (common language effect size = 0.1952) statistical tests are considered weak (small), the Fisher exact test effect size (phi coefficient = 0.5693) is considered strong (moderately high). But no matter which method you prefer, the chance that the entries were randomly duplicated is pragmatically zero.”

**Issue 22:**

*Could there be more interpretation of the effect sizes mentioned, such as Cramer V, common language effect size, and phi coefficient? How do these effect sizes contribute to understanding the strength of the observed relationships?*

**Response:**

We do not want a discussion of effect size to detract from the strongest argument which is impact on model performance as demonstrated in Table 10.

The manuscript already has the following description of the effect sizes:

“While the effect sizes of the chi-squared (Cramer V= 0.2995) and Mann Whitney U (common language effect size = 0.1952) statistical tests are considered weak (small), the Fisher exact test effect size (phi coefficient = 0.5693) is considered strong (moderately high).”

**Issue 23:**

*Should there be more discussion on the pragmatic implications of the findings, especially the assertion that the chance that the entries were randomly duplicated is pragmatically zero? What does this imply for the validity and reliability of the dataset?

Good Luck!*

**Response:**

These are minor points in comparison to the observed drop in model performance as demonstrated in Table 10. Again, we do not want to distract from our main results in Table 10.
